# Impact of LEDGIN treatment during virus production on residual HIV-1 transcription

**DOI:** 10.1186/s12977-019-0472-3

**Published:** 2019-04-02

**Authors:** Gerlinde Vansant, Lenard S. Vranckx, Irena Zurnic, Dominique Van Looveren, Paulien Van de Velde, Christopher Nobles, Rik Gijsbers, Frauke Christ, Zeger Debyser

**Affiliations:** 1 Laboratory for Molecular Virology and Gene Therapy, Department of Pharmaceutical and Pharmacological Sciences, KU Leuven, Herestraat 49, Box 1023, 3000 Leuven, Flanders Belgium; 2 Laboratory for Viral Vector Technology and Gene Therapy, Department of Pharmaceutical and Pharmacological Sciences, KU Leuven, Herestraat 49, Box 1023, 3000 Leuven, Belgium; 3 Department of Microbiology, Perelman School of Medicine, University of Pennsylvania, Philadelphia, USA

**Keywords:** HIV latency, Integrase, LEDGF/p75, LEDGINs

## Abstract

**Background:**

Persistence of latent, replication-competent provirus is the main impediment towards the cure of HIV infection. One of the critical questions concerning HIV latency is the role of integration site selection in HIV expression. Inhibition of the interaction between HIV integrase and its chromatin tethering cofactor LEDGF/p75 is known to reduce integration and to retarget residual provirus to regions resistant to reactivation. LEDGINs, small molecule inhibitors of the interaction between HIV integrase and LEDGF/p75, provide an interesting tool to study the underlying mechanisms. During early infection, LEDGINs block the interaction with LEDGF/p75 and allosterically inhibit the catalytic activity of IN (i.e. the early effect). When present during virus production, LEDGINs interfere with proper maturation due to enhanced IN oligomerization in the progeny virions (i.e. the late effect).

**Results:**

We studied the effect of LEDGINs present during virus production on the transcriptional state of the residual virus. Infection of cells with viruses produced in the presence of LEDGINs resulted in a residual reservoir that was refractory to activation. Integration of residual provirus was less favored near epigenetic markers associated with active transcription. However, integration near H3K36me3 and active genes, both targeted by LEDGF/p75, was not affected. Also in primary cells, LEDGIN treatment induced a reservoir resistant to activation due to a combined early and late effect.

**Conclusion:**

LEDGINs present a research tool to study the link between integration and transcription, an essential question in retrovirology. LEDGIN treatment during virus production altered integration of residual provirus in a LEDGF/p75-independent manner, resulting in a reservoir that is refractory to activation.

**Electronic supplementary material:**

The online version of this article (10.1186/s12977-019-0472-3) contains supplementary material, which is available to authorized users.

## Background

Potent combination antiretroviral therapy (cART) suppresses the plasma viral load of HIV infected patients to undetectable levels. As a result the quality of life has improved significantly and the number of AIDS-related deaths has drastically declined worldwide [1]. Still, globally HIV remains the major cause of death among women between 15 and 49 years old [1]. Moreover, it is challenging from a logistic and economic point of view to provide over 36 million HIV positive patients worldwide with a lifelong treatment [2–4]. In addition, drug related side effects hamper adherence to therapy and allow the emergence of drug resistant HIV strains [5–7]. Therefore, the development of new strategies towards a cure of HIV infection is crucial. The major barrier towards such cure is the persistence of integrated provirus residing mainly in long-lived latently infected memory CD4^+^ T cells responsible for a rebound in viremia upon treatment interruption [8–10]. Although no consensus exists about its relative importance, homeostatic proliferation and clonal expansion of infected cells contribute to viral persistence even under cART [11–17]. Low levels of ongoing viral replication in anatomical sanctuaries with limited drug penetration like the central nervous system, gut associated lymphoid tissue and the lymph nodes may maintain the reservoir as well [18, 19].

Integration of the viral DNA into the host genome is a crucial step in the formation of the proviral reservoir. It is catalyzed by HIV integrase (IN) (For a Review see [20]) with the help of Lens epithelium-derived growth factor (LEDGF/p75). LEDGF/p75 is a transcriptional co-activator that binds IN and tethers the pre-integration complex (PIC) to the chromatin [21–26], facilitating integration into transcriptionally active regions [27]. LEDGF/p75 is not the only determinant of integration site selection [28]. Nuclear import of HIV through nuclear pore complexes (NPC) [29] is a first important step that determines the path through which a PIC enters the nucleus [30, 31]. HIV-1 integration preferentially occurs in the nuclear periphery [32, 33] in active chromatin regions close to the nuclear pore, while inner nuclear or heterochromatic regions are apparently disfavored [34–36]. Depletion of several NPC associated proteins (Nup98, Nup153, Transportin-3, RanBP2 and Tpr) hampers integration in gene dense regions [36–38]. Cleavage and polyadenylation specificity factor 6 (CPSF6) is another cellular cofactor that promotes nuclear entry through its interaction with the HIV capsid protein [39–41]. Depletion or knockout of CPSF6 affects HIV integration in active genes [42–45]. Active genes are characterized by an open chromatin landscape and specific epigenetic histone modifications such as H3K36me3, the recognition mark of LEDGF/p75 [46–48]. Moreover, it was recently found that HIV IN directly interacts with the amino-terminal tail of histone H4, which promotes its anchoring to the nucleosome and facilitates integration [49]. Additionally, HIV IN shows a weak preference for a conserved sequence logo at the site of integration [50–52].

**Fig. 1:**
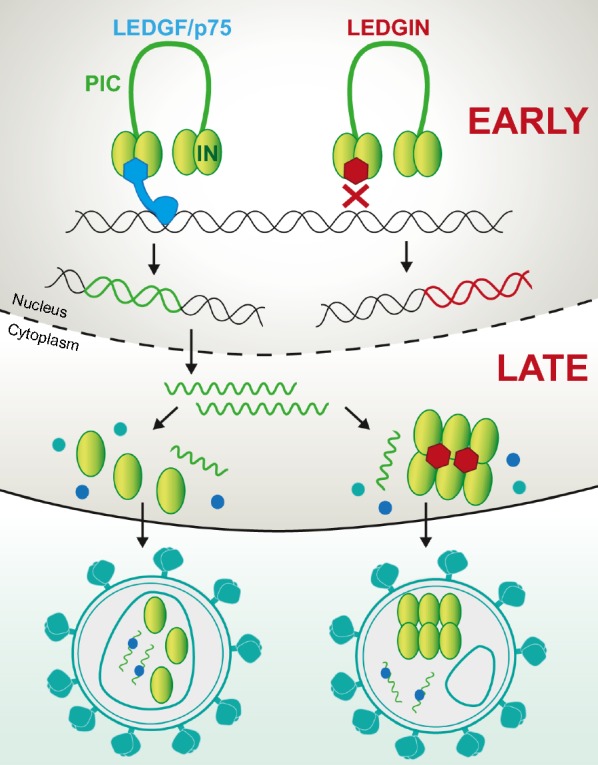
Early and late effects of LEDGINs. LEDGINs inhibit the interaction between the HIV integrase (IN) and the cellular co-factor LEDGF/p75 by binding to the IN dimer interface. This leads to an allosteric inhibition of integration during the early replication steps (early effect; upper panel) [58, 63]. In addition, it relocates integration of residual integrants out of transcription units resulting in more latent provirus [57]. LEDGINs also affect late replication steps; LEDGINs enhance IN oligomerization resulting in maturation defects (late effect; lower panel) [64–67]. These progeny viruses lack the capsid core or the ribonucleoprotein is located outside of the core and are less infectious

As multiple determinants target HIV integration to transcriptionally active regions, the question is raised whether the integration context influences HIV transcription. Previously it was shown that depletion of LEDGF/p75 retargets integration out of active genes [53–57]; these integrants proved to be more transcriptionally silent and refractory to reactivation [57]. Additionally, the LEDGF/p75-IN interaction can be specifically inhibited by small molecule inhibitors [58–61], referred to as LEDGINs [62]. LEDGINs abrogate the binding of LEDGF/p75 to HIV-1 IN by binding to the IN dimer interface and allosterically inhibit the catalytic activity of IN (the so-called ‘early effect’, Fig. 1) [63]. Later it was found that LEDGINs also inhibit late stage replication (the so-called ‘late effect’, Fig. 1) [63–68]. Viral particles produced in the presence of LEDGINs display morphological defects due to LEDGIN-induced IN multimerization [63–67]. Many particles contain a delocalized ribonucleoprotein outside the capsid core or even lack a core. These crippled viruses are hampered during the next round of infection at the level of reverse transcription, nuclear import and integration [63–67]. In 2016, LEDGINs were used as a tool to investigate the link between LEDGF/p75-mediated targeting and the transcriptional state of the provirus. LEDGIN treatment during early replication shifted residual integration out of transcription units in a dose-dependent manner [57]. In addition, the integrated provirus relocalized towards the inner nuclear compartment. Furthermore, the proportion of provirus with a transcriptionally silent phenotype increased, while the reactivation potential was reduced [57]. Of interest, Chen et al. [69] used a barcoded HIV vector to experimentally prove that integration sites affect reactivation of the provirus when stimulated with different LRAs. Moreover, the chromatin functional state of latent HIV provirus also influences latency reversal [70]. Collectively, these results indicate that HIV-1 transcription is affected by integration site selection.

To elaborate this hypothesis, we now investigated the late effect of LEDGINs on integration site selection and HIV-1 expression. In this study, we infected cells with HIV produced in the presence of LEDGINs and determined integration sites and reactivation potential. The fact that LEDGINs inhibit the late replication steps at a lower dose than the early steps [64–68], underscores the translational relevance of this question. LEDGIN treatment during virus production effectively altered immediate quiescence and the activation potential both in cell lines and primary CD4^+^ T cells. Although targeting to H3K36me3 and active genes, were unaffected, the residual integration sites were less favored near features associated with active transcription, reminiscent of a more latent chromatin landscape. These data suggest a LEDGF/p75-independent mechanism generating the silent phenotype observed after LEDGIN treatment during virus production.

## Results

### LEDGIN treatment during virus production reduces infectivity and increases the proportion of quiescent provirus in cell lines

**Fig. 2:**
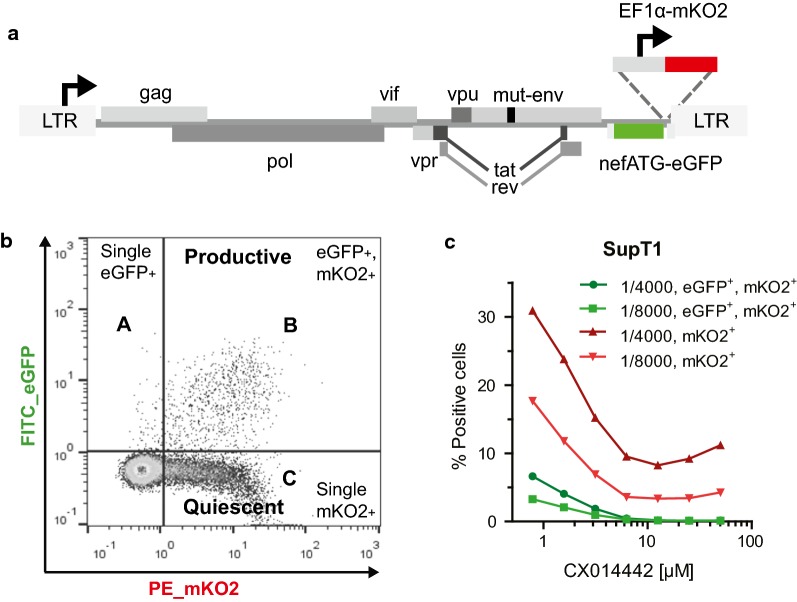
The double reporter virus allows characterization of quiescent and productive infection. **a** Schematic representation of the two-colored OGH reporter virus encoding eGFP in the Nef position driven by the viral LTR promoter and carrying a constitutive transcriptional unit (EF1α-mKO2) inserted downstream [61, 62]. **b** Representative dot plot after flow cytometry analysis of infected SupT1 cells showing the characterization of productive and latent populations based on fluorescence. All infected cells express mKO2 (mKO2^+^). If cells are productively infected, the viral LTR promoter will drive eGFP expression resulting in double positive cells (quadrant B; eGFP^+^, mKO2^+^). Cells only showing mKO2 expression are considered to be quiescently infected (quadrant C). **c** Flow cytometry analysis of infected SupT1 cells (virus dilution 1/4000 and 1/8000) treated with a dilution series of LEDGIN CX014442 during infection. *eGFP* enhanced Green Fluorescent Protein, *mKO2* mutant Kusabira Orange 2

**Fig. 3:**
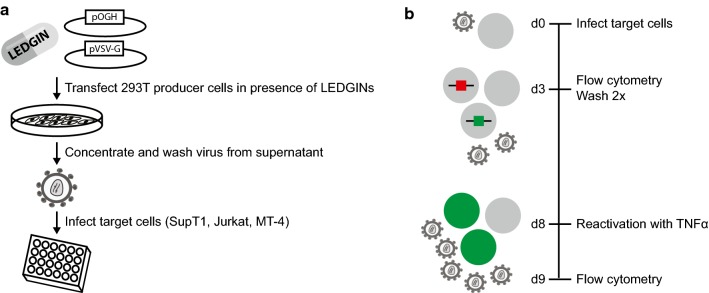
Methodology for virus production and infection experiments. **a** Viruses are produced in HEK293T cells by co-transfection with a plasmid encoding the OGH reporter virus and a plasmid encoding the VSV-G envelope. LEDGINs are added to the cell medium. 72 h post transfection, viruses are harvested from the supernatant, concentrated and washed to remove remaining compound. These viruses can be used to infect different target cells. **b** Different target cells (SupT1, Jurkat, MT-4) were infected with the double reporter virus. Three days post infection (p.i.) samples were taken for flow cytometry and virus was washed away. Cells were reactivated with TNFα eight days p.i. and flow cytometry samples were taken 24 h after reactivation. *TNFα* Tumor Necrosis Factor alpha, *VSV-G* vesicular stomatitis virus G

**Fig. 4:**
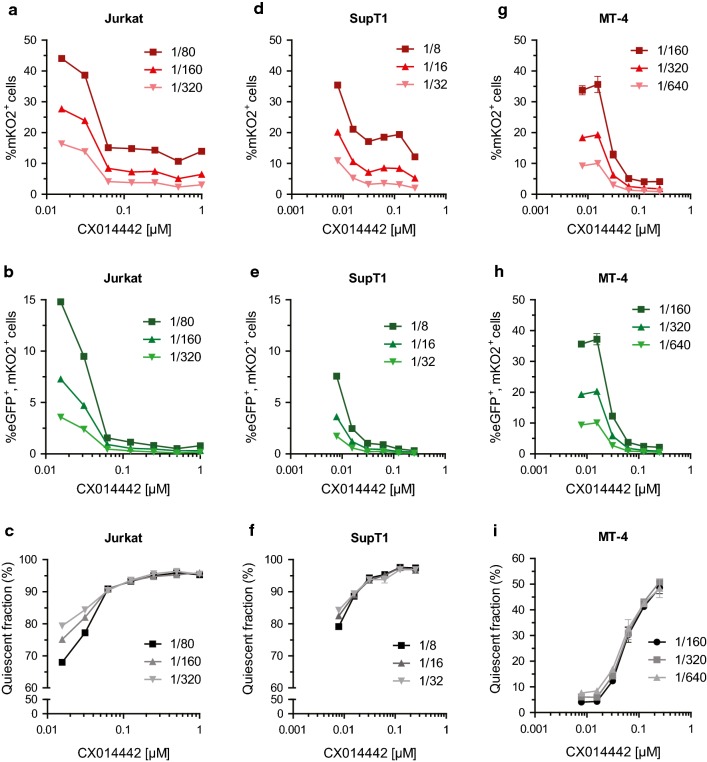
LEDGIN treatment during virus production hampers infectivity and increases the quiescent fraction of residual provirus. Various cell lines were infected with OGH virus produced in the presence of LEDGIN CX014442 (concentrations indicated on x-axes) and analyzed by flow cytometry 3 days post infection. Upper panels: Dose–response curves showing the percentage of total infected mKO2^+^ cells (quadrant B + C, Fig. 2b) with increasing concentration of LEDGIN CX014442. Data represent averages of duplicates with standard deviation from a representative experiment in each cell line. In total five experiments were performed in Jurkat cells, ten in SupT1 cells and three in MT-4 cells. Three different virus dilutions are depicted in various shades of red. Middle panels: Dose–response curve showing the percentage of productively infected (eGFP^+^, mKO2^+^; quadrant B in Fig. 2b) cells with increasing concentration of CX014442. Three different virus dilutions are depicted in shades of green. Lower panels: The quiescent fraction was calculated as the percentage of mKO2 only expressing cells (C/(A + B + C) × 100, Fig. 2b). Data represent averages of duplicates with standard deviation from a representative experiment in each cell line. Three different virus dilutions are depicted in shades of gray. **a**–**c** Dose–response curves for data obtained in Jurkat cells. **d**–**f** Dose–response curves for data obtained in SupT1 cells. **g**–**i** Dose–response curves for data obtained in MT-4 cells. *eGFP* enhanced Green Fluorescent Protein, *mKO2* mutant Kusabira Orange 2

To study the late effect of LEDGINs on HIV transcription, we used a previously described double reporter virus (Fig. 2a) that allows us to simultaneously study silent and productive infection in cell culture [70, 71]. This replication-deficient HIV-1 virus (OGH) encodes for enhanced Green Fluorescent Protein (eGFP) under the control of the viral LTR promoter as well as monomeric Kusabira-Orange2 (mKO2) driven by the constitutively active EF1α promoter. Infected cells carrying an integrated provirus constitutively express mKO2 (Fig. 2b) which is detected by flow cytometry analysis. When the active LTR promoter drives eGFP expression at the same time, resulting in double (mKO2 and eGFP) positive cells, the infection is called productive (quadrant B). Provirus with a silent LTR does not express eGFP and mKO2-only positive cells are referred to as the quiescently infected population (quadrant C). By using this OGH virus, we previously showed that LEDGIN treatment during infection of target cells reduces total (mKO2^+^) and productive infection (eGFP^+^, mKO2^+^) (Fig. 2c). Now we used this double reporter virus to study the effect of LEDGINs on HIV expression when these antivirals are present during virus production (Fig. 3a). Various cell lines (Jurkat, SupT1 and MT-4) were infected with a dilution series of the OGH virus produced in HEK293T cells in the presence of different concentrations of LEDGIN CX014442 (ranging between 7.8 nM to 250 nM for virus used on SupT1 and MT-4 cells, and up to 1 µM for virus used on Jurkat cells) (Fig. 3b). No additional LEDGIN was added during infection of target cells with these viruses. Flow cytometry analysis three days post infection showed a reduced overall infection, as shown by a decrease in percentage of mKO2^+^ cells (quadrant B + C) and in the percentage of productive infection (eGFP^+^, mKO2^+^ cells, quadrant B in Fig. 2b) with increasing concentrations of CX014442 (Fig. 4). The percentage of positive cells obtained in the DMSO control for the different cell lines is shown in Additional file 1: Table S1. We calculated an IC_50_ value of 8.61 ± 0.37 nM in SupT1 cells for the late effect via a nonlinear regression curve fit, while the IC_50_ value of CX014442 added during infection of SupT1 cells was 1.40 ± 0.45 µM. In Jurkat and MT-4 cells, the IC_50_ values of the late effect were 41.62 ± 0.88 nM and 25.54 ± 1.24 nM, respectively. As a control, we repeated the infections in presence of reverse transcriptase inhibitors efavirenz (0.15 µM) and nevirapine (6 µM) to evaluate background signal from non-integrated viral DNA (Additional file 2: Figure S1). Both inhibitors block eGFP and mKO2 expression, proving that there is no plasmid contamination producing fluorescence. These results confirm that infectivity of HIV-1 produced in the presence of LEDGINs is reduced in a concentration dependent manner, as reported before [64, 66, 67].

Next, we investigated whether LEDGIN treatment in producer cells influences HIV expression to a similar extent as previously documented for LEDGIN treatment during infection [57]. Three days post infection, the quiescent fraction was determined as the ratio of single mKO2 positive cells over the total number of detected eGFP and mKO2 positive cells (C/(A + B + C) × 100, Fig. 2b). With increasing concentrations of CX014442 an augmentation in the quiescent fraction from about 70-80% up to 97% was observed in both Jurkat and SupT1 cells (Fig. 4c, f). In MT-4 cells, the quiescent fraction increased from less than 10% in the DMSO condition (Additional file 1: Table S1) to 50% with virus produced in the presence of 0.25 µM of CX014442 (Fig. 4i). The quiescent fraction calculated for the DMSO control for the different cell lines is shown in Additional file 1: Table S1. We conclude that LEDGIN treatment during virus production reduces infection of target cells in a dose-dependent manner and that residual integrants are more often in a transcriptionally silent state.

### LEDGIN treatment during virus production results in a quiescent reservoir refractory to reactivation

**Fig. 5:**
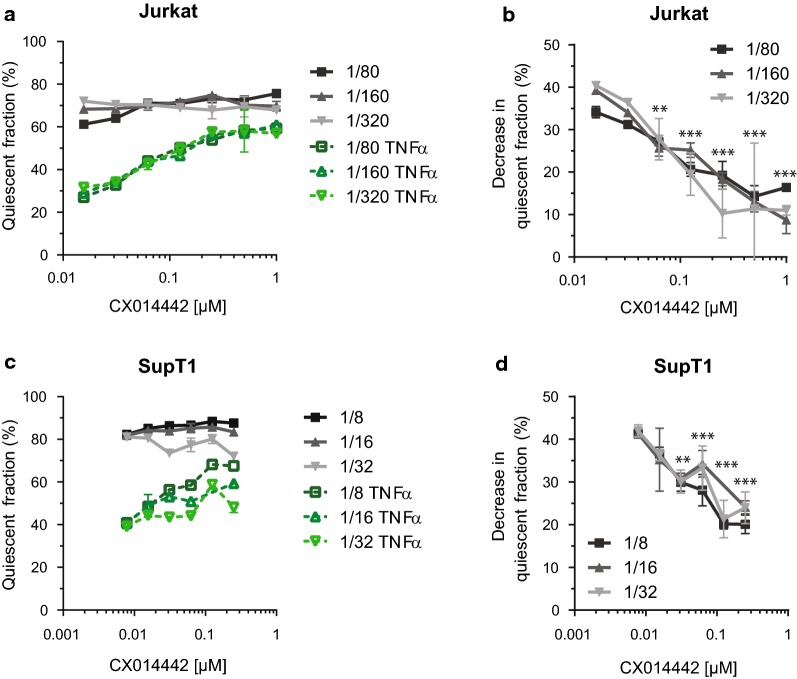
LEDGIN treatment during virus production hampers reactivation of residual integrants. Eight days post infection with OGH virus produced in presence of LEDGINs, cells were reactivated in duplicate with 10 ng/µl TNFα for 24 h followed by flow cytometry analysis. **a**, **c** The quiescent fraction (C/(A + B + C) × 100, Fig. 2b) is depicted for unactivated cells in gray and activated cells in green for three different virus dilutions in Jurkat (**a**) and SupT1 cells (**c**). **b**, **d** The decrease in quiescent fraction between non-activated and TNFα-treated cells is shown for three different virus dilutions in Jurkat (**b**) and SupT1 cells (**d**). Data represent averages of duplicates with standard deviation from a representative experiment (total five experiments in Jurkat cells and ten in SupT1 cells). Statistical significance was determined for all dilutions at each concentration of LEDGINs compared to the DMSO control via a one-way ANOVA with Dunnet’s multiple comparison test (**p < 0.01, ***p < 0.001). *TNFα* Tumor Necrosis Factor alpha, *DMSO* dimethyl sulfoxide

We further characterized this quiescent provirus by evaluating its reactivation potential. Jurkat cells and SupT1 cells were infected with a dilution series of the OGH virus that was produced in the presence of different concentrations of CX014442. Cells were reactivated with tumor necrosis factor α (TNFα) 8 days post infection (Fig. 3b). 24 h after reactivation, we performed flow cytometry analysis (Additional file 2: Figure S2). Reactivation of cells resulted in an increased percentage of eGFP expressing cells (Additional file 2: Figure S2b, d) and a decrease in the percentage of single mKO2^+^ quiescent cells (Fig. 5a, c), implying a shift from quiescent (Fig. 2b, quadrant C) to productive infection (quadrant B). The quiescent fraction (C/(A + B + C) × 100) in the DMSO condition decreased with 40% upon reactivation (Additional file 1: Table S1), whereas prior treatment with 1 µM of CX014442 yielded a decrease that was less than 10% in Jurkat cells (Fig. 5b). The same phenotype was observed in SupT1 cells (Fig. 5d). This decrease was statistically significant (p < 0.0001) as demonstrated by a one-way ANOVA with Dunnett’s multiple comparison test that compared all values at each CX014442 concentration with those of the DMSO control. We also performed reactivation 13 days post infection of SupT1 cells to assure stable latency establishment, and obtained similar results (Additional file 2: Figure S3). Additionally, we evaluated different latency reversing agents (LRAs) in SupT1 cells (Additional file 2: Figure S4). We compared 10 ng/ml TNFα with 3 µM phorbol myristate acetate (PMA), 5 µM prostratin and 1 µM suberoylanilide hydroxamic acid (SAHA). TNFα induced the strongest stimulation, however when using other LRAs, we consistently observed less reactivation in cells infected with virus that was produced in the presence of 1 µM of CX014442 compared to the DMSO (0 µM) control. These results indicate that the residual reservoir in cell lines, formed after infection with HIV particles produced in the presence of LEDGINs, is refractory to HIV reactivation. MT-4 cells were not included because more than 90% of infected cells in the DMSO control were productive and therefore it was difficult to achieve additional stimulation with TNFα.

### The chromatin environment of residual provirus after LEDGIN treatment during virus production is associated with latency

**Fig. 6:**
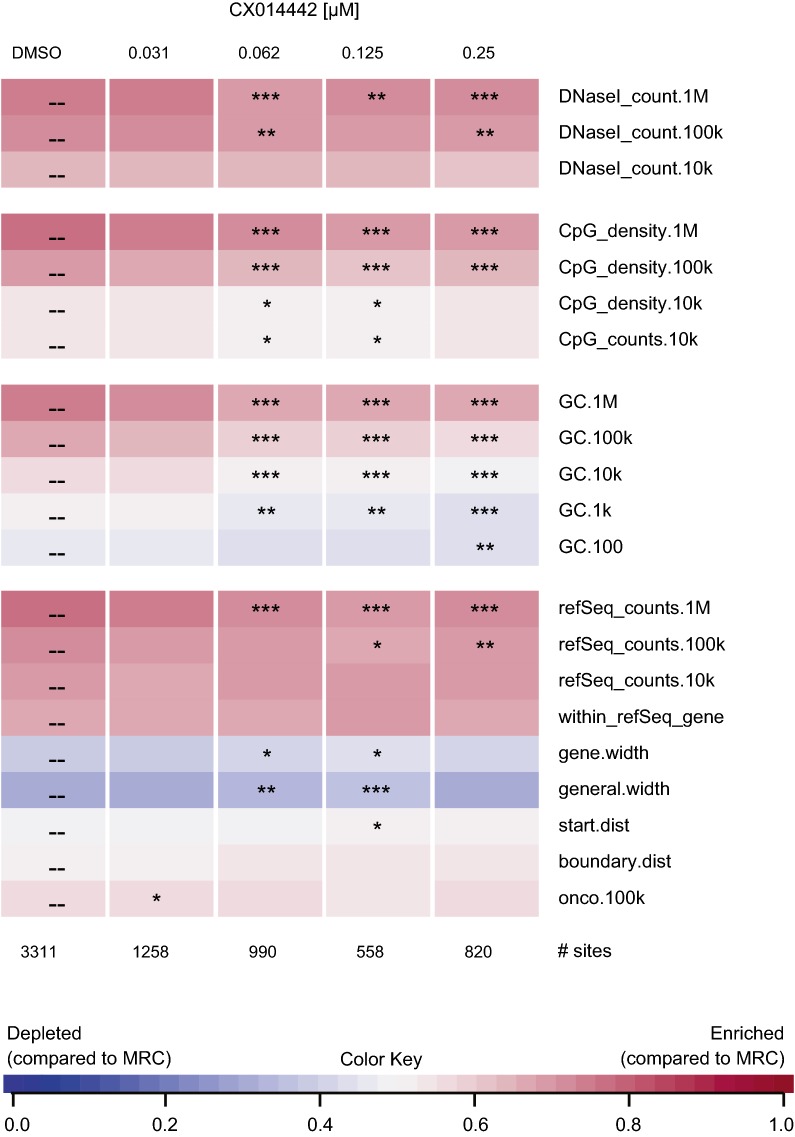
LEDGIN treatment during virus production does not affect targeting to refSeq genes. SupT1 cells were transduced with a 1/20 dilution of an HIV vector that was produced in the presence of a dilution series of LEDGIN CX014442. The presence of various genomic features near integration sites was determined using the INSPIIRED software (Bushman lab, University of Pennsylvania). The heat map summarizes information on integration sites for the different concentrations of LEDGIN (columns) and different genomic features (rows). Tile colors indicate whether integration is favored (pink) of disfavored (blue) near a certain genomic feature compared to matched random control sites using a receiver operating characteristic (ROC) curve area. The ROC curve area scale is shown below. Statistical significance (asterisks, ranked Wald tests) is shown relative to the DMSO data set (*p < 0.05, **p < 0.01, ***p < 0.001). *DMSO* dimethyl sulfoxide

**Fig. 7:**
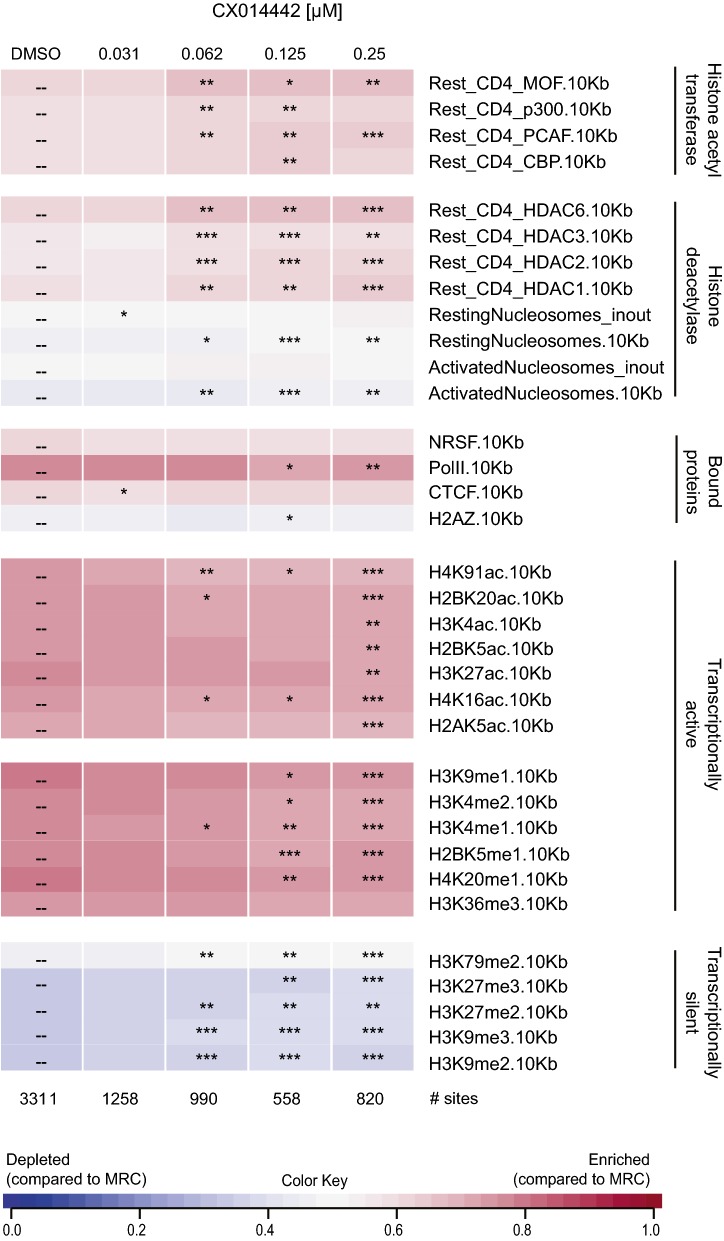
Chromatin environment of residual provirus after LEDGIN treatment during virus production is associated with latency. SupT1 cells were transduced with a 1/20 dilution of an HIV vector that was produced in the presence of a dilution series of LEDGIN CX014442. The presence of various epigenetic features near integration sites was determined using the INSPIIRED software (Bushman Lab, University of Pennsylvania). The heat map summarizes information on integration sites for the different concentrations of LEDGIN (columns) and different epigenetic features (rows). Tile colors indicate whether integration is favored (pink) of disfavored (blue) near a certain epigenetic feature compared to matched random control sites using a receiver operating characteristic (ROC) curve area. The ROC curve area scale is shown below. Statistical significance (asterisks, ranked Wald tests) is shown relative to the DMSO data set (*p < 0.05, **p < 0.01, ***p < 0.001). *DMSO* dimethyl sulfoxide

LEDGIN treatment during infection was recently found to affect integration site distribution with an impact on transcription of the provirus [57]. Here we investigated whether the quiescent phenotype of residual provirus after production in the presence of LEDGINs is associated with an altered integration site distribution. SupT1 cells were transduced with an eGFP expressing lentiviral vector that was produced in the presence of a dilution series of LEDGIN CX014442. Flow cytometry confirmed a dose-dependent inhibition of transduction by CX014442 treatment during production of the vector (Additional file 2: Figure S5). Integration site analysis of cells transduced with a 1/20 vector dilution is represented as a genomic heat map (Fig. 6) that plots whether a certain genomic feature is (dis)favored near the integration site compared to matched random control sites [72]. This correlation is visualized by the color scale: favored features relative to matched random control sites (MRC) are plotted in pink, while blue represents features that are disfavored compared to MRC. Statistical significance calculated by the Wald test is shown for all LEDGIN conditions relative to the DMSO data set (Fig. 6). Consistent with previously published data [27, 57, 73–75], integration was favored in refSeq genes (76.43%) in the absence of inhibitor (Additional file 1: Table S2). CX014442 treatment in producer cells did not significantly affect integration in refSeq genes (‘within_refSeq_gene’, Fig. 6 and Additional file 1: Table S2). Still, the occurrence of refSeq genes within a distance of 100 kb and 1 Mb of the integration site was significantly lower compared to the DMSO control (‘refSeq_counts’, Fig. 6). Furthermore, integration was somewhat less favored near DNaseI sensitive sites, CpG islands and in GC rich regions when producer cells were treated with CX014442 compared to the DMSO control. Next, sequencing data were analyzed for various epigenetic features, resulting in an epigenetic heat map (Fig. 7). In line with earlier reports [57], for the DMSO control, integration preferentially occurred near markers for active transcription (e.g. H4K91ac, H4K16ac, H3K4me1, H3K4me2), while integration in transcriptionally silent regions (e.g. H3K9me3, H3K27me3) was disfavored. Addition of CX014442 during vector production resulted in a distinct chromatin environment that was characterized by less active transcription markers and an enrichment in markers for transcriptionally silent regions (Fig. 7). Remarkably, integration near H3K36me3, the epigenetic feature recognized by LEDGF/p75, was not altered upon addition of CX014442 during vector production. Similar results were obtained for SupT1 cells transduced with a 1/40 dilution of the vector (Additional file 2: Figures S6 and S7). These data indicate that LEDGIN treatment during vector production results in a more latent chromatin landscape of the residual provirus.

### LEDGIN treatment inhibits integration and HIV activation in primary CD4^+^ T cells

**Fig. 8:**
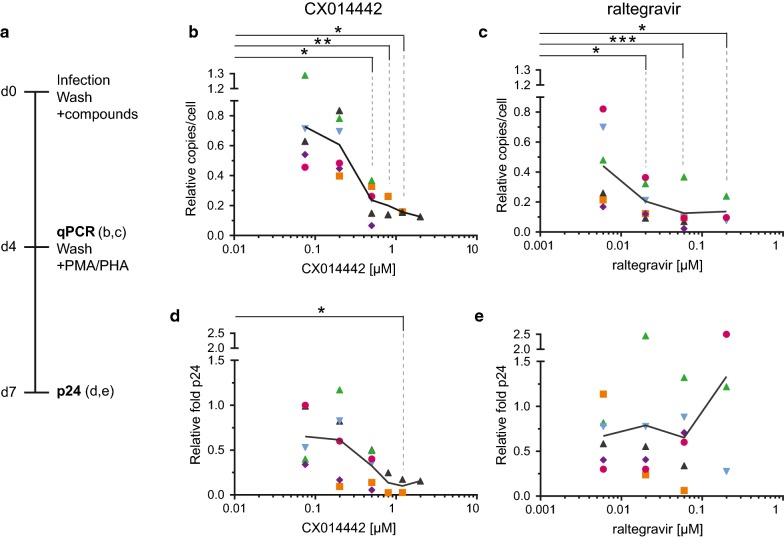
LEDGIN treatment in primary CD4^+^ T cells hampers reactivation. **a** Primary CD4^+^ T cells from six independent donors were infected with wild type NL4.3 virus in presence of LEDGIN CX014442 or raltegravir. Four days post infection samples for qPCR were harvested, remaining cells were washed and activated with 10 nM PMA and 10 µg/ml PHA. Viral p24 was measured in the supernatant 7 days post infection. Results for six independent donors are represented by different dots and the average is shown by the connecting line. All results were plotted relative to the DMSO control, which was set to ‘1’ in each experiment. **b** The number of integrated copies per cell with increasing concentration of CX014442, as determined 4 days post infection. **c** The number of integrated copies 4 days after infection in the presence of raltegravir. **d** 72 h after activation, the fold activation of cells treated with CX014442 was calculated as the ratio of viral p24 in the supernatant of activated cells compared to non-activated cells. **e** The fold increase in p24 upon activation of cells treated with raltegravir. Statistical significance was calculated by the Kruskal–Wallis test that compared each concentration of compound with the DMSO control (*p < 0.05, **p < 0.01, ***p < 0.001). *DMSO* dimethyl sulfoxide, *PMA* phorbol 12-myristate 13-acetate, *PHA* phytohaemagglutinin

To evaluate the effect of LEDGINs on activation in a more relevant setting, experiments in primary CD4^+^ T cells were performed. In these experiments LEDGIN treatment was compared with raltegravir, a classical IN strand transfer inhibitor on the market. Primary CD4^+^ T cells were isolated from fresh buffy coats from six healthy donors and infected with wild type NL4.3 virus in the presence of different concentrations of LEDGIN CX014442 (0.075–2 µM) or raltegravir (0.006–0.2 µM). This experimental set up allows multiple rounds of viral replication, resulting in a combined early and late effect of LEDGINs when added during the infection. Four days after infection, compounds and virus were washed away and cells were activated (Fig. 8a). Both IN inhibitors significantly reduced the number of integrated copies per cell when measured 4 days after infection as determined by the Kruskal–Wallis test (CX014442: p = 0.0009, raltegravir: p = 0.0009) (Fig. 8b, c, Additional file 1: Table S3). Next, we evaluated relative activation by determining the fold increase in viral p24 levels in cell supernatants 72 h after stimulation with 10 µg/ml phytohaemagglutinin (PHA) and 10 nM phorbol myristate acetate (PMA). The combination of PHA and PMA was used in all donors as this combination stimulated our cells the most (Additional file 2: Figure S8). Cells without inhibitor treatment displayed at least a six-fold increase in p24 production upon stimulation (Additional file 1: Table S3). For the donor cells with the strongest activation we observed a 44-fold increase in viral p24. Because of the considerable donor-to-donor variation, all values were normalized to the DMSO control that was set to ‘1’ in each experiment. CX014442 treatment during infection significantly (p = 0.0079) hampered activation in a dose-dependent manner (Fig. 8d), whereas cells treated with raltegravir were equally activated in these experiments (Fig. 8e). Remarkably, although LEDGIN was added during infection of the CD4^+^ T cells, submicromolar concentrations of the compound (0.5 µM) were sufficient to generate this phenotype. In addition to these experiments, we also performed infections with WT NL4.3-eGFP virus in the presence of LEDGINs, to provide an additional flow cytometry readout that detects eGFP expressing infected cells. This readout is complementary to the p24 measurement, as p24 does not provide information on the number of cells containing virus. Four days after infection, the percentage of eGFP positive cells (between 0.3 and 1.7%, measured for at least 100,000 cells) corresponded well with the number of integrated copies detected via qPCR (between 0.2 and 2 copies/100 cells) (Additional file 2: Figure S9a, b). Next, these cells were activated with PMA and PHA for 72 h and analyzed by p24 ELISA and flow cytometry (Additional file 2: Figure S9c, d). The percentage of eGFP positive cells in the DMSO condition increased from about 3 up to 20% upon stimulation. Finally, for both readouts we observed less activation after treatment with increasing concentration of CX014442 (Additional file 2: Figure S9e, f).

## Discussion

The latent HIV reservoir is the main target for different HIV cure strategies [76]. The so-called shock-and-kill strategy aims to reactivate latent provirus with latency reversing agents (LRAs) and subsequent killing of the reactivated cells by viral cytopathic effects or by immune clearance [76–81]. So far results are hampered by insufficient potency and toxicity of latency reversing agents (LRA) [82–85]. It was shown that LRAs reactivate less than 5% of latent provirus [70] and that the response to different LRAs depends on the site of integration [69]. This might explain the limited success of the shock-and-kill strategy. Therefore, it remains important to explore other cure strategies. Recently, a novel block-and-lock strategy was proposed that aims at locking the residual virus into a ‘deep’ latent or transcriptionally silent state lacking the capability to rebound upon cART cessation [57, 76, 86–92]. This deep latent state can be achieved in several ways. HIV transcription can be abrogated by inhibition of trans-activator of transcription (Tat) [86–88]. Alternatively, HIV expression and reactivation can be inhibited by LEDGIN-mediated retargeting of HIV integration to sites that are less susceptible to reactivation [57]. To achieve an HIV cure via any of the discussed strategies, it is important to understand the role of integration site selection in HIV latency and reactivation. In this study, we further explored this relation by investigating the late effect of LEDGINs on residual HIV integration and expression.

LEDGINs were added during virus production (late effect), resulting in crippled progeny virions with an enhanced IN oligomerization [64–67]. First, we confirmed that LEDGIN treatment in producer cells reduces infectivity of the progeny virus in the next round of infection when using a dual colored reporter virus. These results are in full agreement with previously published data on the late effect of LEDGINs [64–67]. Next, we evaluated whether LEDGIN treatment during virus production alters HIV expression. After treatment with 0.25 µM LEDGIN CX014442 97% of residual provirus in Jurkat and SupT1 cells was in a quiescent state and less susceptible to reactivation. In the DMSO control only 70–80% of infected cells were quiescent. This result phenocopies the effect seen with LEDGIN treatment during infection [57]. In MT-4 cells the quiescent fraction was also augmented with increasing concentration of CX014442. Whereas in the DMSO condition less than 10% of provirus was quiescent, treatment with 0.25 µM of LEDGIN increased the proportion of silent provirus up to 50%. Notably, the extent of eGFP expression in the DMSO control samples varied among cell lines due to different activation of the LTR promoter. This emphasizes the importance of comparing latency and reactivation phenotypes in multiple cell lines in parallel. The MT-4 cell line is transformed with human T cell lymphotropic virus type I (HTLV-1), activating the host T cell [93]. Since MT-4 cells already constitute an active cell line, it is more difficult to achieve additional activation. In fact, we found that more than 90% of the MT-4 cells in the DMSO control were productively infected, and therefore MT-4 cells were not included in the reactivation experiments.

Next, we investigated whether retargeting of integration sites can explain the quiescent phenotype observed with viruses produced in the presence of LEDGINs. LEDGF/p75 is the main tethering factor guiding HIV to active transcription units [27] and depletion of LEDGF/p75 or disruption of its interaction with HIV IN is known to shift integration out of active genes [53–57]. Interestingly, CX014442 treatment during virus production did not significantly alter the percentage of integration in refSeq genes or near H3K36me3, the recognition mark of LEDGF/p75, in contrast to integration sites obtained with CX014442 treatment during early infection (Additional file 1: Table S2, data from [57]). The crippled viruses likely still depend on LEDGF/p75 for integration. When comparing integration sites after early or late LEDGIN treatment a similar pattern is observed (Additional file 1: Table S4). In both cases, integration becomes less favored near DNaseI, CpG islands and active transcription markers, while integration near transcriptionally silent markers is enriched upon addition of LEDGIN CX014442. Integration sites obtained after CX014442 treatment during virus production or during early infection of cells are both less favored in GC rich regions, although the effect is more pronounced when CX014442 is added during production. Overall, it seems that the main difference lies in targeting to refSeq genes. The IC_50_ value for the late effect of LEDGIN CX014442 is much lower, only up to 0.25 µM of compound was added during production. For the early effect, concentrations from 0.78 up to 50 µM were used (Additional file 1: Table S2). Higher concentrations of LEDGIN are thus required to shift integration out of refSeq genes. Changes in the chromatin environment on the other hand, already occur at the low LEDGIN concentrations used during production. Since these low concentrations are sufficient to cause the latent phenotype, the data suggest that a mechanism other than pure retargeting by LEDGF/p75 is involved. This is in agreement with our previous observation that the LEDGIN induced quiescent phenotype is stronger than the mere effect on retargeting [57]. Enhanced IN oligomerization is a common feature seen both for early and late effect of LEDGINs and might play a role in this latent phenotype. Using Förster resonance energy transfer (FRET) we previously demonstrated a LEDGF/p75-dependent increase in IN multimerization in the nucleus [94]. Upon knockdown of LEDGF/p75, this phenotype was rescued by addition of LEDGINs during infection of the cells [94]. Moreover, LEDGIN treatment during virus production enhances IN oligomerization prematurely in the viral particle, and these multimers are retained in the cytoplasm and nucleus after infection of target cells [64, 94]. Although enhanced/premature IN oligomerization may not affect targeting by LEDGF/p75, it might still influence in which chromatin environment integration takes place, for instance by a direct interaction between IN and histone amino-terminal tails [49]. The exact mechanism remains to be clarified.

Finally, our data obtained in primary CD4^+^ T cells validate the use of LEDGINs in a more clinically relevant acute infection model. LEDGIN CX014442 hampered WT HIV-1 activation in a dose-dependent manner. Although CX014442 was added during infection, the IC_50_ value obtained when using WT HIV-1 (237 ± 0.28 nM) was 26-fold lower compared to the IC_50_ value for single round infection of primary cells with the OGH virus (6.21 ± 0.22 µM) [57]. This is due to the combination of the early and late effect of LEDGINs during multiple round replication with WT HIV-1. During multiple round infection in the presence of LEDGINs most likely a combination of LEDGF/p75-dependent and independent effects on integration site selection occur. How the observed effects possibly translate into patients and whether LEDGINs can be used in a block-and-lock functional cure needs to be investigated in advanced latency cell models, humanized mouse models and eventually in clinical trials.

## Conclusion

Altogether, our data provide additional evidence for a link between integration site selection and HIV expression. LEDGIN treatment during virus production resulted in a residual reservoir that was more often in a quiescent state and refractory to activation. Integration was less favored in transcriptionally active chromatin, however, still mainly occurred in refSeq genes. In contrast to the quiescent phenotype seen upon LEDGIN treatment during early infection which is LEDGF/p75-dependent [57], we now observe a LEDGF/p75-independent phenotype. Possibly LEDGIN-enhanced IN oligomerization interferes with proper integration site selection. Our research shows that LEDGINs are a useful tool to investigate the importance of integration site selection and provide a rationale to further study their effects in context of a future block-and-lock cure strategy.

## Materials and methods

### Cell culture and virus production

All cells were verified to be mycoplasma free by a cellular colorimetric detection assay (PlasmoTest™, InvivoGen Europe). Cells were cultured at 37 °C in a humidified atmosphere containing 5% CO_2_. SupT1 cells (provided by the National Institutes of Health reagent program, NIH, Bethesda, MD) [95, 96] were cultured in RPMI medium (GIBCO BRL) with 10% (v/v) fetal bovine serum (FBS, GIBCO) and 0.01% (v/v) gentamicin (GIBCO). HEK293T cells (generous gift from O. Danos, Evry, France) were cultured in Dulbecco Modified Eagle Medium (DMEM, GIBCO, Dublin, Ireland) with 5% (v/v) fetal bovine serum (FBS, GIBCO) and 0.01% v/v gentamicin (GIBCO). HEK293T cells were co-transfected, using linear polyethylenimine (PEI, Polysciences), with a plasmid encoding a single round HIV virus (pOGH) [70, 71] and a vesicular stomatitis virus G (VSV-G) protein encoding plasmid to produce VSV-G-pseudotyped viruses (Fig. 3a). Cells were washed twice with Phosphate Buffered Saline (PBS) to remove the excess of plasmid and the medium was replaced by medium containing different concentrations (7.8 nM–1 µM) of LEDGIN CX014442 [63] 6 h post transfection. The supernatant was collected 72 h post transfection and filtered through a 0.45 µm pore membrane (Merck, Overijse, Belgium). The virus was concentrated using a Vivaspin with a 15–50 kDa cut-off column (Merck) and washed three times with PBS. Next, the virus was treated with 100 U/ml DNase (Roche Diagnostics, Vilvoorde, Belgium) for 1 h at 37 °C to eliminate remaining plasmid and stored at − 80 °C. The vector used for integration site sequencing was produced by triple transfection with the transfer plasmid pCH-SFFV-eGFP-P2A-fLuc together with the Δ8.91 packaging plasmid and pVSV-G.

### Reporter virus

A multi-colored reporter virus (OGH) (Fig. 2a) was used to study the late effect of LEDGINs. This green-orange variant of the recently described LAI-based double reporter virus [70, 71] contains an LTR-driven enhanced Green Fluorescent Protein (eGFP) in the Nef gene position and a constitutively active EF1α promotor driving mutant Kusabira-Orange 2 (mKO2) expression [70]. Simultaneous flow cytometry measurement of both reporters allows characterization of quiescent and active provirus (Fig. 2b) [97, 98]. Infected cells that are exclusively mKO2 positive, due to its expression driven by the internal constitutive promotor, are considered to comprise the LTR-silent or quiescent proviral pool. Cells expressing both mKO2 and eGFP are considered productively infected, as LTR-driven transcription is activated in these cells.

### Reactivation experiments in cell lines

300,000 cells (Jurkat, SupT1 and MT-4 cells) were infected for 3 days in a 48-well plate with different dilutions of OGH virus that was produced in the presence of LEDGIN CX014442 as described above. The viral stocks were normalized on their p24 content (Innotest HIV antigen mAb, Fujirebio Europe). 72 h post infection, cells were washed twice with PBS and reseeded in a 12-well plate (Fig. 3b). At day eight, cells were reactivated in duplicate using 10 ng/ml Tumor Necrosis Factor α (TNFα, Immunosource, Zoersel, Belgium). Flow cytometry was performed on samples taken 3 days after infection and on day nine, 1 day after reactivation.

### Flow cytometry analysis

Fluorescence was measured after cells were fixed in 2% paraformaldehyde (PFA) for 15 min at room temperature (RT) using a MACS Quant VYB analyzer (Miltenyi Biotech GmbH, Bergisch Gladbach, Germany). To measure eGFP expression, cells were excited using a 488 nm, 50 mW DPSS (diode-pumped solid-state) laser and the emitted signal passed through a 525/50 nm band pass filter. For mKO2 expression a 561 nm, 100 mW diode laser and 586/15 nm band pass filter were used. Live cells were selected based on the forward and side scatter channel (FSC-H/SSC-H) and doublets were excluded based on the FSC-A/FSC-H plot. For experiments in cell lines, at least 25,000 single live cells were counted in total and each sample was measured in duplicate. Single reporter constructs were used as controls. For flow cytometry analysis of primary CD4^+^ T cells infected with WT NL4.3-eGFP virus, at least 100,000 cells were counted. Data were analyzed using the FlowJo software (FlowJo LCC, Ashland, Oregon).

### Integration site sequencing

Integration sites were determined as described previously [99]. 100,000 SupT1 cells were transduced for 3 days with a lentiviral vector (CH-SFFV-eGFP-P2A-fLuc) that was produced in the presence of LEDGIN CX014442. Next, they were washed twice with PBS and kept in culture for at least 10 days to eliminate non-integrated DNA. Genomic DNA was extracted using the QIAamp DNA Mini kit (Qiagen). Genomic DNA was randomly sheared by sonication with the Covaris M220 and linkers were added to the sheared DNA ends. Integration sites were amplified by nested PCR using primers complementary to the linker and viral long terminal repeats (LTR). PCR products were sequenced by Illumina Miseq, paired-end 300 cycles. The INSPIIRED software [72] was used to analyze sequencing data.

### Isolation of resting CD4^+^ T-cells

Human peripheral blood mononuclear cells (PBMC) were isolated from fresh buffy coats obtained from the Red Cross Blood transfusion Center (Mechelen, Belgium) using a lymphoprep density gradient centrifugation (Stem cell technologies, Cologne, Germany). Resting CD4^+^ T cells were enriched using a custom-made Easysep negative selection kit (Stem Cell Technologies, #19052 with the addition of CD25, CD69, and HLA-DR antibodies) and magnetic beads (Stem Cell Technologies), resulting in a purity of 95%. The experiments with human blood cells received bioethical approval by the Medical Ethics committee of the KU Leuven (S58969-IRB00002047).

### Infection and activation of primary CD4^+^ T-cells

Freshly isolated resting CD4^+^ T cells were activated with 100 U/ml IL-2 (Peprotech, London, UK) and 10 µg/ml phytohaemagglutinin (PHA, Sigma) 48 h before infection. Cells were infected with wild type (WT) NL4.3 or WT NL4.3-eGFP virus (1.6 × 10^6^ ng p24 per 1 × 10^7^ cells/mL) for 3 h at 37 °C. Next, the excess of virus was washed away with PBS (three times) and cells were resuspended in RPMI medium with 10% (v/v) FBS and 0.1% gentamicin, supplemented with 1 U/ml IL-2 (Peprotech) and varying concentrations of LEDGIN CX014442 or raltegravir (provided by National Institute of Health AIDS reagent program, NIH, Bethesda, MD). Four days post infection compounds and virus were again washed away with PBS (three times). Cells were replated and activated with 10 nM phorbol myristate acetate (PMA, Sigma) and 10 µg/ml PHA or left untreated. Activation was quantified by measuring the viral p24 concentration in the culture supernatant 7 days post infection (Innotest HIV antigen mAb, Fujirebio Europe) and by flow cytometry for cells infected with WT NL4.3-eGFP virus. Four days post infection cells were harvested to determine the number of integrated copies using Alu-LTR qPCR and via flow cytometry for cells infected with WT NL4.3-eGFP.

### Quantification of total integrated copy number

Integrated HIV DNA was quantified using a nested real-time Alu-LTR qPCR [100]. 1 million cells were lysed in 50 µl of lysis buffer for 1 h at 56 °C (10 mM Tris HCl pH8, 1 mM EDTA, 0.01% triton and 0.8 mg/ml Proteinase K (PK)). The first round PCR reaction mix consisted of 5 µl of DNA from lysed cells, 12.5 µl of iQ supermix (Bio rad, Temse, Belgium), 0.5 µl of each primer (20 µM, Alu forward: TCCCAGCTACTGGGGAGGCTGAGG, Alu reverse: TGCTGGGATTACAGGCGTGAG and HIV-1 LTR forward: GCTAACTAGGGAACCCACTGCTTA) and 6 µl of water. Cycling conditions for the first round PCR were 95 °C for 10 min, followed by 15 cycles of 95 °C for 30 s, 60 °C for 40 s and 72 °C for 3.5 min. All samples were run at least in duplicate. 5 µl of the first-round product was added to a second round PCR mix containing 12.5 µl of iQ supermix, 0.5 µl of forward and reverse primer (20 µM, HIV-1 LTR forward: AGCTTGCCTTGAGTGCTTCAA, HIV-1 LTR reverse: TGACTAAAAGGGTCTGAGGGATCT), 1 µl of probe (5 µM, 5′-FAM-TTACCAGAGTCACACAACAGACGGGCA-TAMRA-3′) and 5.5 µl of water. Second round PCR was performed in a LightCycler 480 (Roche Life Science, Vilvoorde, Belgium) for 5 min at 95 °C, followed by 45 cycles of 95 °C for 15 s, 60 °C for 30 s and 72 °C for 1 min. As a standard genomic DNA of SupT1 cells transduced with the OGH virus and passaged for 3 weeks, was used. Integrated copies were normalized for input DNA by a parallel CCR5 qPCR as previously described [101]. Data were analyzed using the provided LightCycler 480 software.

### Statistical analysis

All data was analysed using the GraphPad Prism software version 7.00 for Windows (GraphPad Software, La Jolla California USA). IC_50_ values were calculated via a nonlinear regression curve fit of the concentration of inhibitor versus response. The statistical significance of the effect of LEDGINs compared to the control sample was assessed via one-way ANOVA with Dunnett’s multiple comparisons test in cell lines or with the Kruskal–Wallis test for primary cell data.

## Additional files

**Additional file 1.** Additional tables.

**Additional file 2.** Additional figures.
