# Supplementary material for: Impact of LEDGIN treatment during virus production on residual HIV-1 transcription
Source: Retrovirology. 2019 Apr 2;16:8. doi: 10.1186/s12977-019-0472-3 (PMC6444612; doi:10.1186/s12977-019-0472-3)
Supplement: Supplementary file 1 — Additional file 1. Additional tables. [file 12977_2019_472_MOESM1_ESM.pdf]

Table S1: Flow cytometry data of the DMSO control in different cell lines.

Jurkat	1/80	1/160	1/320
%mKO2$^+$	27.40	17.00	9.43
%eGFP$^+$, mKO2$^+$	7.44	3.94	2.05
Quiescent fraction (%)	74.35	77.87	79.19
Decrease in quiescent fraction (%)	38.04	37.50	39.00
**SupT1**	**1/8**	**1/16**	**1/32**
%mKO2$^+$	46.5	30.35	16.65
%eGFP$^+$, mKO2$^+$	12.10	6.25	3.06
Quiescent fraction (%)	74.56	78.99	82.08
Decrease in quiescent fraction (%)	42.12	40.10	41.66
**MT-4**	**1/160**	**1/320**	**1/640**
%mKO2$^+$	30.56	16.64	8.45
%eGFP$^+$, mKO2$^+$	28.10	14.65	6.94
Quiescent fraction (%)	5.40	6.89	9.47

Three days post infection with OGH virus produced in the presence of DMSO, the percentage of total infected cells (mKO2+; quadrant B+C in Figure 2b), productively infected cells (eGFP+, mKO2+; quadrant B in Figure 2b) and the quiescent fraction was determined. Eight days post infection, cells were reactivated with TNFα for 24 h causing a decrease in quiescent fraction. Results are shown for Jurkat, SupT1 and MT-4 cells for three different virus dilutions. DMSO; dimethyl sulfoxide, eGFP; enhanced Green Fluorescent Protein, mKO2; mutant Kusabira Orange 2, TNFα; Tumor Necrosis Factor alpha.

Table S2: Integration in refSeq genes is not affected by LEDGIN treatment in producer cells.

	CX014442 [µM]	# sites	% in refSeq genes	
Late effect	DMSO	3311	76.43	
	0.031	1258	74.67	
	0.062	990	77.59	
	0.125	558	78.67	
	0.25	820	74.67	
	**CX014442 [µM]**	**# sites**	**% in refSeq genes**	
Early effect	DMSO	3312	69.54	
	0.78	2451	70.58	
	1.56	2278	70.37	
	3.12	2485	66.60	
	6.25	3365	65.23	**
	12.5	884	61.88	***
	25	604	60.93	***
	50	418	54.55	***

SupT1 cells were transduced with a 1/20 dilution of an HIV-1 vector produced in the presence of a dilution series of LEDGIN CX014442. The upper part of the table gives a summary of the number of unique integration sites retrieved for the different conditions and the corresponding percentage of integrations in refSeq genes (late effect). The lower part of the table shows results previously obtained for LEDGIN treatment during transduction (early effect) of SupT1 cells (1).

Table S3: Raw data for experiments in primary CD4+ T cells.

		[µM]	Donor 1	Donor 2	Donor 3	Donor 4	Donor 5	Donor 6
Integrated copies per 1000 cells	CX014442	DMSO	0.57 (0.20)	10.35 (0.78)	1.40 (0.36)	1.17 (0.18)	15.38 (2.62)	26.86 (7.94)
		0.075	0.26 (0.08)	5.60 (0.39)	1.00 (0.19)	1.50 (0.20)		16.91 (0.75)
		0.2	0.28 (0.07)	4.63 (3.17)	0.98 (0.16)	0.91 (0.23)	6.12 (2.23)	22.41 (1.65)
		0.5	0.15 (0.05)	0.68 (0.86)	0.34 (0.24)	0.43 (0.02)	5.04 (0.13)	4.01 (0.60)
		0.8					4.02 (0.32)	3.74 (0.45)
		1.2					2.44 (1.55)	4.17 (0.72)
		2						2.54 (0.62)
	Raltegravir	DMSO	0.57 (0.20)	10.35 (0.78)	1.40 (0.36)	1.17 (0.18)	15.38 (2.62)	26.86 (7.94)
		0.006	0.47 (0.24)	1.74 (0.02)	0.98 (0.17)	0.56 (0.08)	3.30 (1.82)	5.57 (0.68)
		0.02	0.21 (0.07)	1.24 (0.00)	0.30 (0.12)	0.38 (0.02)	1.89 (0.33)	6.98 (0.63)
		0.06	0.05 (0.03)	0.23 (0.07)	0.15 (0.15)	0.43 (0.14)	1.46 (0.14)	2.48 (0.41)
		0.2	0.06 (0.03)	0.19 (0.02)	0.11 (0.06)	0.28 (0.04)		1.87 (0.17)
Fold increase in p24	CX014442	DMSO	6.22	6.09 (1.66)	12.12 (0.61)	8.75 (0.88)	44.61 (3.51)	19.59 (2.43)
		0.075	6.14	2.06 (0.28)	6.40 (5.16)	3.50 (0.23)		19.39 (5.27)
		0.2	3.96	1.02 (0.01)	10.00 (0.19)	10.25 (0.34)	4.18 (0.13)	16.08 (1.95)
		0.5	2.31	0.33 (0.08)	4.25 (0.97)	4.32 (0.30)	6.17 (0.24)	9.87 (0.11)
		0.8					1.12 (0.17)	4.83 (0.75)
		1.2					1.14 (0.06)	3.39 (0.36)
		2						3.03 (0.42)
	Raltegravir	DMSO	6.22	6.09 (1.66)	12.12 (0.61)	8.75 (0.88)	44.61 (3.51)	19.58 (2.43)
		0.006	1.80	2.45 (0.21)	9.39 (0.94)	7.16 (0.32)	50.69 (5.14)	7.13
		0.02	2.16	2.47 (0.18)	9.40 (2.33)	21.45 (13.9)	10.53 (0.27)	11.42
		0.06	3.96	4.30 (0.64)	10.65 (0.20)	11.55 (3.30)	2.77 (0.18)	10.84 (0.44)
		0.2	15.40		3.31 (0.46)	10.66 (3.53)		6.64 (0.46)

Upper part: the average number of integrated copies per 1000 cells and standard deviation (gray), determined four days after infection, is shown for all conditions in each donor. Lower part: the average fold increase in concentration of p24 and standard deviation is determined 72 h after stimulation with PMA and PHA and plotted for each condition for the different donors. PMA; phorbol 12-myristate 13-acetate, PHA; phytohaemagglutinin

Table S4: Summary integration site sequencing data

Feature	Late effect	Early effect (data from (1))
refSeq genes	Not changed	Less favored (-)
GC content	Less favored (- -)	Less favored (-)
DnaseI	Less favored (-)	
CpG islands	Less favored (-)	
Active transcription markers	Less favored (-)	
Silent transcription markers	More favored (+)	

Summarizing table comparing integration site sequencing data obtained for LEDGIN treatment during virus production (Late effect) and LEDGIN treatment during infection of cells (Early effect, data from (1)).

Reference

1. Vranckx LS, Demeulemeester J, Saleh S, Boll A, Vansant G, Schrijvers R, et al. LEDGIN-mediated Inhibition of Integrase–LEDGF/p75 Interaction Reduces Reactivation of Residual Latent HIV. EBioMedicine [Internet]. 2016 Jun 13;8:248–64. Available from: http://www.ncbi.nlm.nih.gov/pmc/articles/PMC4919729/
