# Supplementary material for: Impact of LEDGIN treatment during virus production on residual HIV-1 transcription
Source: Retrovirology. 2019 Apr 2;16:8. doi: 10.1186/s12977-019-0472-3 (PMC6444612; doi:10.1186/s12977-019-0472-3)
Supplement: Supplementary file 2 — Additional file 2. Additional figures. [file 12977_2019_472_MOESM2_ESM.pdf]

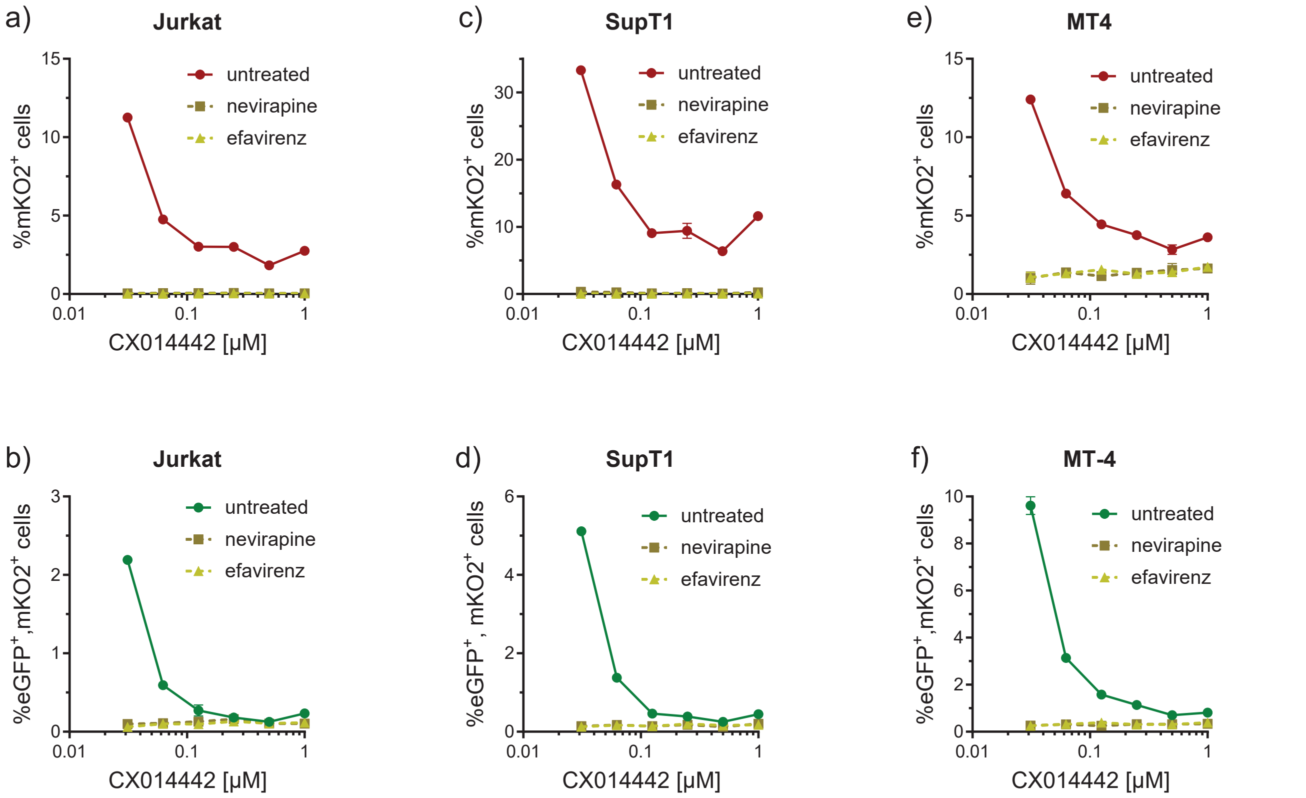

Figure S1: The percentage of positive cells 3 days after infection in the presence of reverse transcriptase inhibitors. Efavirenz (0.0015 µM) or nevirapine (0,06 µM) were added during infection of different cell lines with OGH virus that was produced in the presence of LEDGINs. Dose-response curves showing the percentage of positive cells with increasing concentration of LEDGIN CX014442 three days after infection for untreated cells, cells treated with nevirapine (brown) or efavirenz (yellow). Data represent averages of duplicates with standard deviation from an experiment in Jurkat (a,b), SupT1 (c,d) and MT-4 cells (e,f). (a,c,e) Dose-response curve for the percentage of total infected (mKO2+) cells. (b,d,f) Dose-response curve for the percentage of productively infected (eGFP+,mKO2+) cells. eGFP; enhanced Green Fluorescent Protein, mKO2; mutant Kusabira Orange 2.

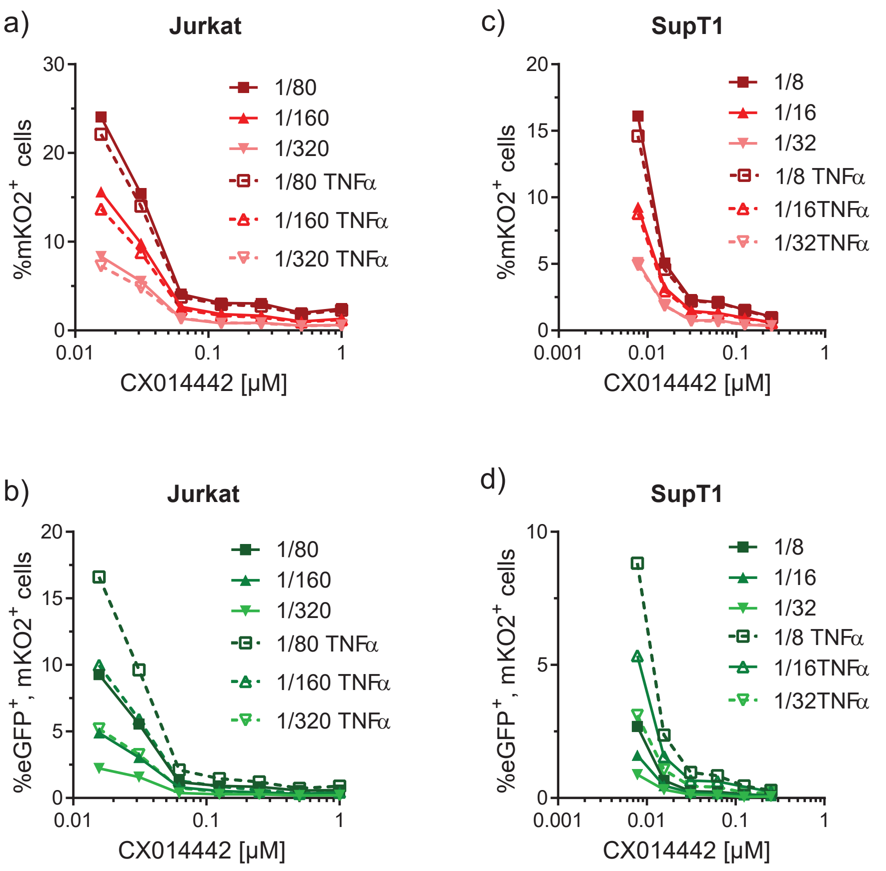

Figure S2: The percentage of positive cells 9 days after infection with OGH virus produced in the presence of LEDGINs. Dose-response curves showing the percentage of positive cells with increasing concentration of LEDGIN CX014442 nine days after infection. Three different virus dilutions are depicted in solid lines for the untreated cells and in dashed lines for cells activated with TNFα. Data represent averages of duplicates with standard deviation from a representative experiment in Jurkat (a,b) and SupT1 cells (c,d). (a,c) Dose-response curve for the percentage of total infected (mKO2+) cells. (b,d) Dose-response curve for the percentage of productively infected (eGFP+, mKO2+) cells. TNFα; Tumor Necrosis Factor alpha, eGFP; enhanced Green Fluorescent Protein, mKO2; mutant Kusabira Orange 2.

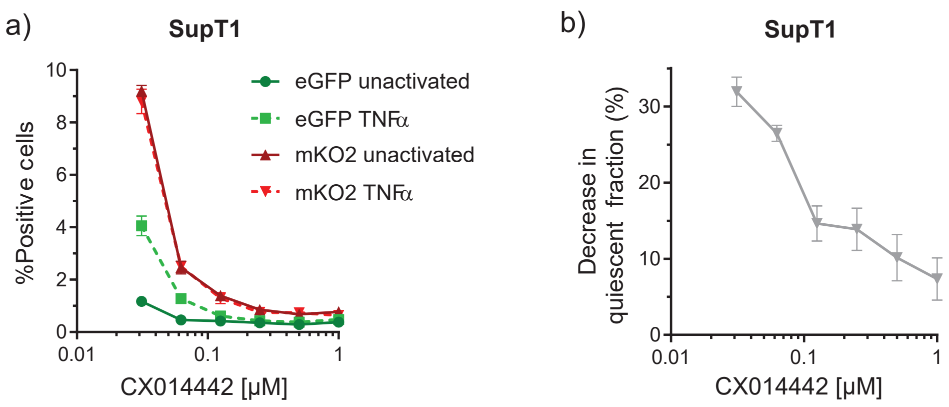

Figure S3: Reactivation of SupT1 cells 13 days post infection with OGH virus produced in the presence of LEDGINs. SupT1 cells were infected with OGH virus that was produced in the presence of different concentrations of LEDGIN CX014442. 13 days post infection (p.i.) cells were reactivated with TNFα. (a) Dose-response curves showing the percentage of positive cells with increasing concentration of CX014442 14 days after infection. Untreated cells are plotted by solid lines and cells activated with TNFα in dashed lines. Data represent averages of duplicates with standard deviation from a representative experiment in SupT1 cells. (b) Reactivation is plotted as the decrease in quiescent fraction upon treatment with TNFα. TNFα; Tumor Necrosis Factor alpha, eGFP; enhanced Green Fluorescent Protein, mKO2; mutant Kusabira Orange 2.

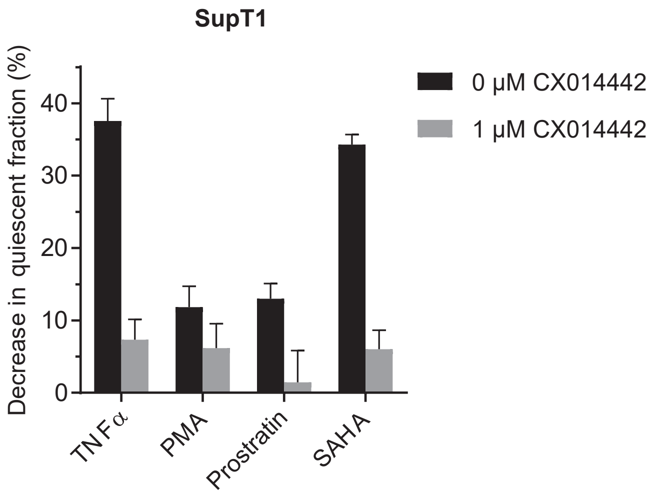

Figure S4: Comparison of different LRAs in SupT1 cells. SupT1 cells were infected with OGH virus that was produced in the presence of 0 µM or 1 µM of LEDGIN CX014442. Eight days post infection (p.i.) cells were reactivated with different LRAs for 24 h. Reactivation is plotted as the decrease in quiescent fraction upon stimulation. LRA; Latency Reversing Agent, TNFα; Tumor Necrosis Factor alpha 10 ng/ml, PMA; phorbol 12-myristate 13-acetate 3 µM, prostratin 5 µM, SAHA; suberoylanilide hydroxamic Acid 1 µM, eGFP; enhanced Green Fluorescent Protein, mKO2; mutant Kusabira Orange 2.

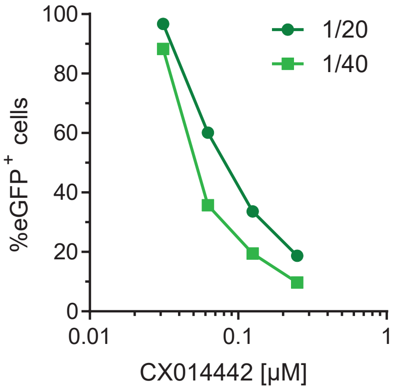

Figure S5: LEDGIN treatment during vector production hampers infectivity. SupT1 cells were transduced with an HIV-1 vector that was produced in the presence of a dilution series of LEDGIN CX014442. The percentage of eGFP+ cells 12 days after transduction decreases with increasing concentration of compound. Results are shown for two different vector dilutions. eGFP; enhanced Green Fluorescent Protein.

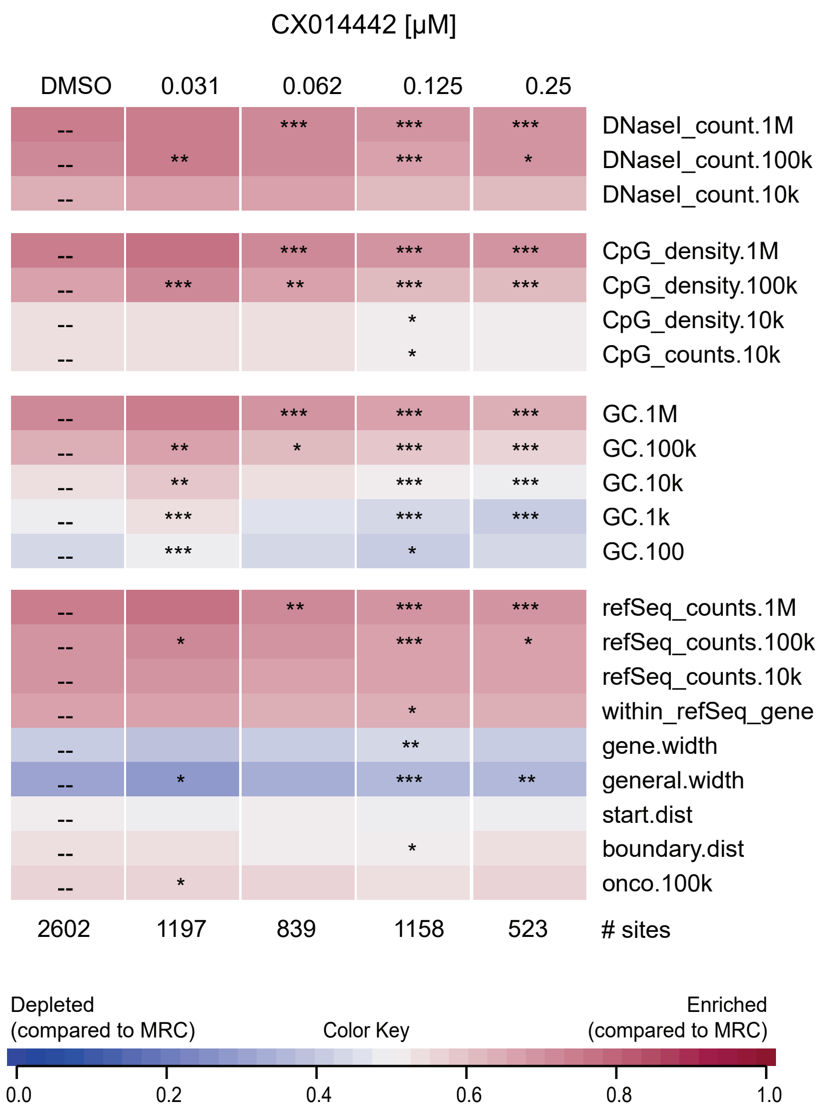

Figure S6: Genomic heat map of integration sites obtained after transduction with a 1/40 dilution of an HIV-1 vector produced in the presence of LEDGINs. SupT1 cells were transduced with a 1/40 dilution of an HIV-1 vector produced in the presence of a dilution series of LEDGIN CX014442. The presence of various genomic features near integration sites was determined using the INSPIIRED software (Bushman lab, University of Pennsylvania). The heat map summarizes information on integration sites for the different concentrations of LEDGIN (columns) and different genomic features (rows). Tile colors indicate whether integration is favored (pink) or disfavored (blue) near a certain genomic feature compared to matched random control sites using a receiver operating characteristic (ROC) curve area. ROC curve area scale is shown at the bottom. Statistical significance (asterisks, ranked Wald tests) is shown relative to the DMSO data set (*p<0.05, **p<0.01, ***p<0.001). DMSO; dimethyl sulfoxide.

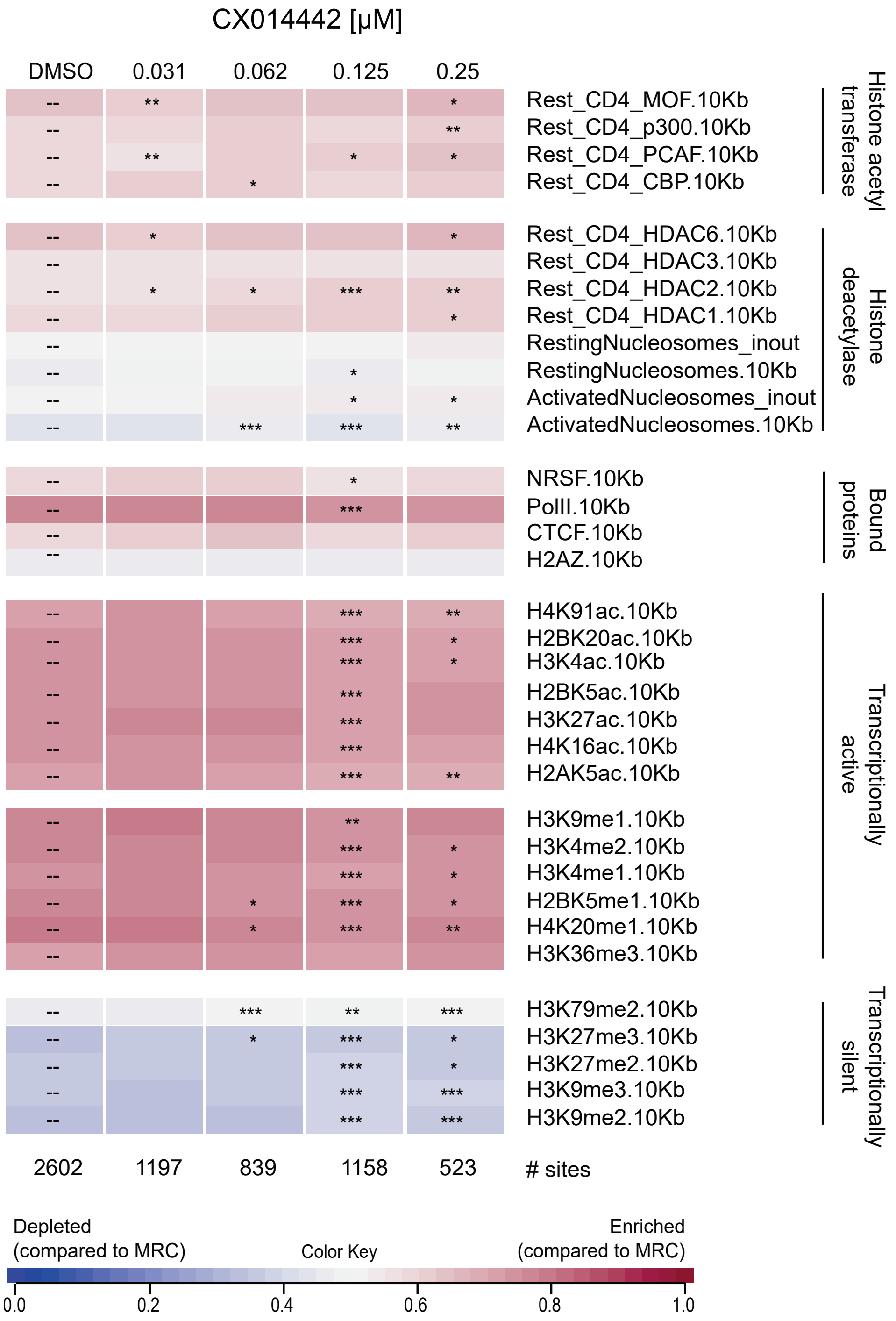

Figure S7: Epigenetic heat map of integration sites obtained after transduction with a 1/40 dilution of an HIV-1 vector produced in the presence of LEDGINs. SupT1 cells were transduced with a 1/40 dilution of a lentiviral vector produced in the presence of a dilution series of LEDGIN CX014442. The presence of various epigenetic features near integration sites was determined using the INSPIIRED software (Bushman lab, University of Pennsylvania). The heat map summarizes information on integration sites for the different concentrations of LEDGIN (columns) and different epigenetic features (rows). Tile colors indicate whether integration is favored (pink) of disfavored (blue) near a certain epigenetic feature compared to matched random control sites using a receiver operating characteristic (ROC) curve area. ROC curve area scale is shown at the bottom. Statistical significance (asterisks, ranked Wald tests) is shown relative to the DMSO data set (dashes)(*p<0.05, **p<0.01, ***p<0.001). DMSO; dimethyl sulfoxide.

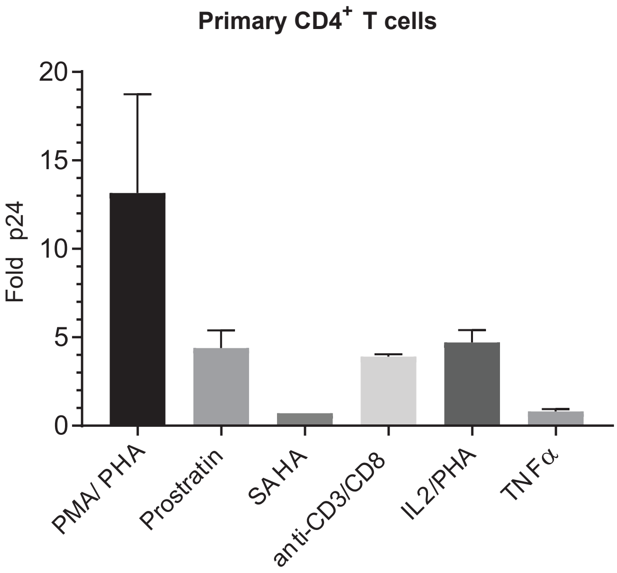

Figure S8: Comparison of different LRAs in primary CD4+ T cells. Four days after infection with WT NL4.3 virus, primary CD4+ T cells were washed and activated with different stimuli. After 72 h of activation, viral p24 was determined in the supernatant. Reactivation is expressed as the ratio of p24 levels in activated cells on p24 levels in untreated cells. Results are plotted as the average with standard deviation of two independent donors. PMA/PHA; phorbol 12-myristate 13-acetate 10 nM and phytohaemagglutinin 10 µg/ml, prostratin 10 µM, SAHA; suberoylanilide hydroxamic acid 3 µM, IL2/PHA; interleukin 2 100 U/ml and PHA 10 µg/ml, TNFα; Tumor Necrosis Factor alpha 10 ng/ml.

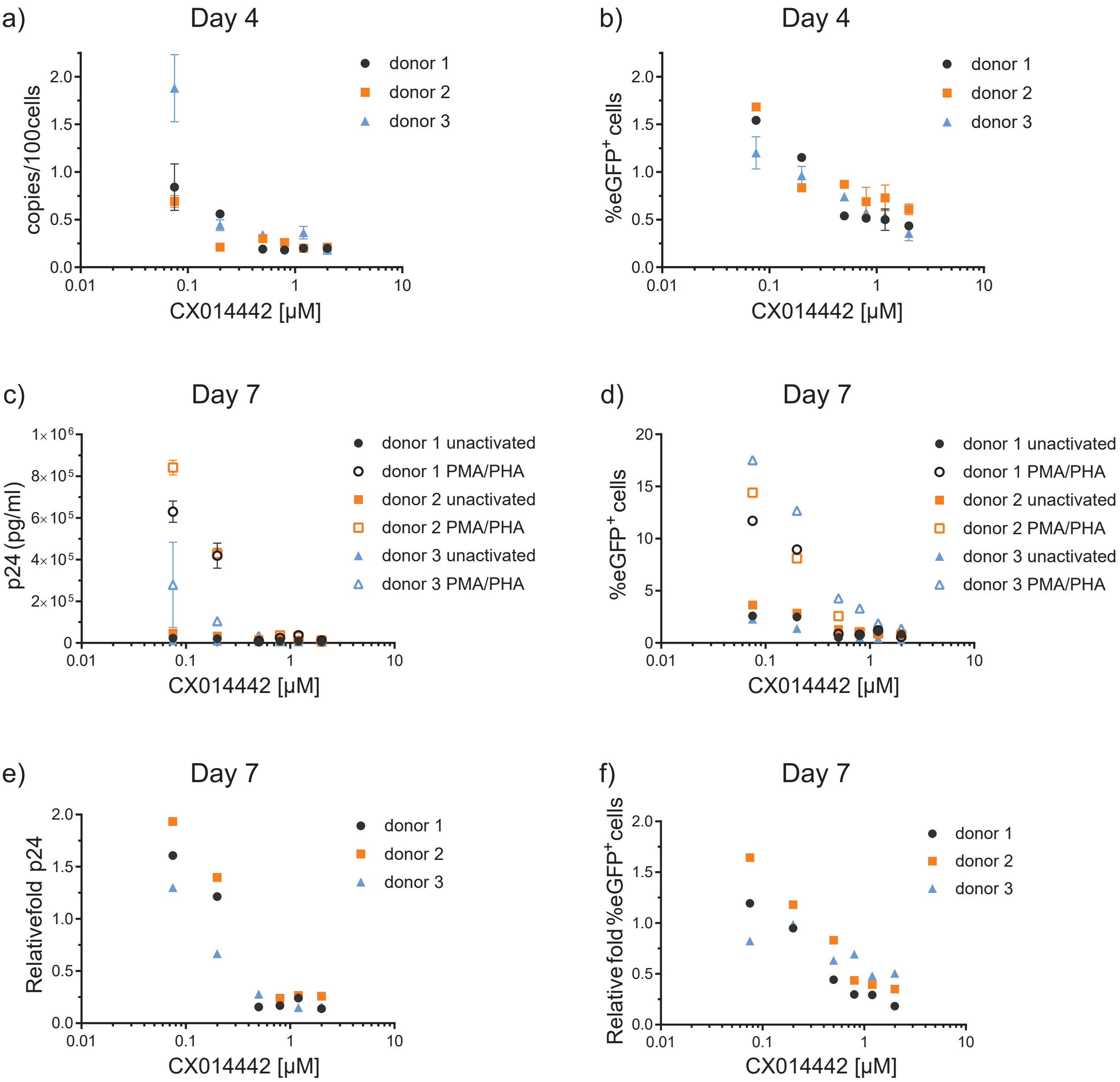

Figure S9: Infection of primary CD4+ T cells with WT NL4.3-eGFP virus in presence of LEDGINs. Primary CD4+ T cells of three independent donors were infected with WT NL4.3-eGFP virus in the presence of different concentrations of CX014442. (a,b) Four days post infection (p.i.) cells were analyzed via qPCR and flow cytometry. (c,d) Next, cells were washed and activated with 10 nM PMA and 10 µg/ml PHA. On day 7 p.i. viral p24 was determined in the supernatant and cells were analyzed by flow cytometry. (e,f) Reactivation is expressed as the fold increase in p24 or %eGFP+ positive cells of activated cells compared to unactivated cells. The fold increase is plotted relative to the DMSO control which was set to ‘1’. Results of three independent donors are plotted by a different symbol and color. PMA; phorbol 12-myristate 13-acetate, PHA; phytohaemagglutinin, eGFP; enhanced Green Fluorescent Protein, DMSO; dimethyl sulfoxide.
